# Supplementary material for: The Oral Health of a Group of 19th Century South Australian Settlers in Relation to Their General Health and Compared with That of Contemporaneous Samples
Source: Dent J (Basel). 2023 Apr 7;11(4):99. doi: 10.3390/dj11040099 (PMC10136771; doi:10.3390/dj11040099)
Supplement: Supplementary file 1 [file dentistry-11-00099-s001.zip › Supplementary_Table S1. Dental Inventory_Dental & Skeletal Age Ranges_Sex_.pdf]

The oral health of a group of 19th Century South Australian settlers in relation to their general health and compared with that of contemporaneous samples. Gurr et al., 2023.

Supplementary Material Table S1. St Mary's Cemetery - Dental inventory.

**St Mary's Burial I/D**	**Sex**	**†Dental age range** (months/years) London Atlas of human tooth development and eruption (AlQahtanti et al., 2010)	**Skeletal age range** (years) (Anson, 2004)	Number of primary and/ or permanent teeth present	**Dental Inventory** Tooth type present. ***FDI** notation number/s
**SMB 66 A**	U	7 mths-1 (+/- 3 mths)	0-2	6 *primary*	51, 52, 61, 62, 71, 81
**SMB 40**	U	10 mths-1 (+/- 3 mths)	0-2	6 *primary*	51, 61, 62, 71, 81, 82
**SMB 32**	U	10.5 mths-1 (+/- 3 months)	0-2	8 *primary*	51, 52, 61, 62, 71, 72, 81,82
**SMB 41**	U	1-1.5 (+/- 3 mths)	0-2	5 *primary*	54, 62, 64, 71, 81
**SMB 08**	U	1-1.5 (+/- 3 mths)	0-2	8 *primary*	51, 54, 61, 64, 71, 81, 82, 84
**SMB 62**	U	1-1.5 (+/- 3 mths)	0-2	10 *primary*	51, 52, 61, 64, 71, 72, 74, 81, 82, 84
**SMB 82**	U	1-1.5 (+/- 3 mths)	0-2	9 *primary*	51, 52, 54, 61, 62, 71, 74, 81, 84
**SMB 24**	U	1-1.5 (+/- 3 mths)	0-2	9 *primary*	51, 52, 54, 61, 62, 64, 74, 82, 84
**SMB 27 B**	U	1-1.5 (+/- 3 mths)	0-2	10 *primary*	51, 52, 54, 61, 71, 72, 74, 81, 82, 84
**SMB 58**	U	1-1.5 (+/- 3 mths)	0-2	11 *primary*	51, 52, 61, 62, 64, 71, 72, 74, 81, 82, 84
**SMB 18**	U	1-1.5 (+/- 3 mths)	0-2	1 *primary*	74
**SMB 11**	U	2-2.5 (+/- 3 mths)	0-2	19 *primary*	51, 52, 53, 54, 55, 61, 62, 63, 64, 71, 72, 73, 74, 75, 81, 82, 83, 84, 85
**SMB 31**	U	3-3.5 (+/- 6 mths)	3-5	17 *primary*	51, 53, 54, 55, 63, 64, 65, 71, 72, 73, 74, 75, 81, 82, 83, 84, 85
**SMB 04A**	U	3.5-4.5 (+/- 6 mths)	3-5	19 *primary*	51, 52, 53, 54, 55, 61, 62, 63, 64, 71, 72, 73, 74, 75, 81, 82, 83, 84, 85
**SMB 75**	U	5-6 (+/-1 yr)	6-9	14 *primary*	54, 55, 62, 63, 64, 65, 72, 73, 74, 75, 81, 83, 84, 85
**SMB 35**	U	6-7 (+/-1 yr)	6-9	11 *primary*	51, 52, 54, 63, 64, 65, 73, 74, 75, 84, 85
**SMB 19**	U	7.5-8.5 (+/-1 yr)	6-9	7 *primary* 15 permanent	11, 12, 13, 14, 15, 16, 21, 22, 24, 65, 26, 32, 73, 74, 75, 36,41, 42, 83, 84, 85, 46,
**SMB 70**	U	11.5-12.5 (+/-1 yr)	6-9	2 *primary* 6 permanent	53, 14, 15, 21, 63, 24, 25, 26
**SMB 52 B**	U	10.5-11.5 (+/-1 yr)	10-15	2 *primary* 16 permanent	11, 12, 13, 16, 21, 22, 23, 26, 31, 32, 33, 75, 36, 41, 42, 43, 85, 46
**SMB 51**	U	10-11 (+/-1 yr)	10-15	1 primary 15 permanent	12, 13, 14, 15, 16, 22, 23, 24, 25, 26, 31, 34, 36, 41, 42, 84,
**SMB 28**	F	15.5-16.5 (+/-1 yr)	10-15	26 permanent	11, 12, 14, 15, 16, 17, 22, 23, 24, 25, 26, 27, 31, 32, 33, 34, 35, 36, 37, 41, 42, 43, 44, 45, 46, 47
**SMB 79**	U	15.5-16.5 (+/-1 yr)	16-19	25 permanent	11, 13, 14, 15, 17, 21, 22, 23, 24, 25, 26, 27, 31, 32, 33, 34, 35, 37, 41, 42, 43, 44, 45, 46, 47
**SMB 05**	F	Over 23.5	20-29	6 permanent	32, 35, 41, 42,43, 44,
**SMB 53 C**	F	Over 23.5	30-39	9 permanent	11, 13, 14, 21, 23, 26, 34, 42, 46
**SMB 66 B**	F	Over 23.5	30-39	17 permanent	12, 14, 15, 17, 21, 22, 23, 27, 31, 32, 33, 34, 41, 42, 43, 44, 45
**SMB 73**	M	Over 23.5	30-39	19 permanent	11, 12, 13, 16, 21, 22, 23, 25, 28, 31, 32, 33, 34, 35, 41, 42, 43, 44, 45

**SMB 06**	M	Over 23.5	40-49	24 permanent	11, 12, 13, 14, 15, 16, 17, 22, 23, 24, 25, 26, 27, 31, 32, 33, 37, 41, 42, 43, 44, 45, 46, 47
**SMB 09**	M	Over 23.5	40-49	22 permanent	12, 13, 16, 21, 22, 23, 24, 25, 26, 31, 32, 33, 34, 35, 36, 37, 43, 44, 45, 46, 47, 48
**SMB 57**	M	Over 23.5	40-49	25 permanent	11, 12, 13, 14, 15, 16, 17, 18, 22, 26, 27, 32, 33, 34, 35, 36, 37, 41, 42, 43, 44, 45, 46, 47, 48,
**SMB 61**	F	Over 23.5	40-49	7 permanent	25, 32, 33, 35, 41, 42, 44
**SMB 72**	M	Over 23.5	40-49	29 permanent	11, 12, 13, 14, 15, 16, 17, 18, 21, 22, 23, 24, 25, 26, 27, 32, 33, 34, 35, 36, 37, 38, 42, 43, 44, 45, 46, 47, 48
**SMB 78**	M	Over 23.5	40-49	4 permanent	13, 14, 34, 35
**SMB 83**	M	Over 23.5	40-49	16 permanent	13, 14, 17, 21, 23, 24, 27, 28, 33, 34, 37, 38, 42, 43, 47, 48
**SMB 85**	M	Over 23.5	40-49	2 permanent	33, 34
**SMB 14**	M	Over 23.5	50-59	2 permanent	23, 35
**SMB 23**	M	Over 23.5	50-59	21 permanent	13, 14, 15, 17, 18, 21, 22, 23, 27, 28, 31, 32, 33, 34, 35, 37, 41, 42, 43, 44, 47
**SMB 59**	M	Over 23.5	50-59	15 permanent	11, 12, 13, 21, 22, 23, 34, 35, 37, 38, 41, 42, 44, 46, 48
**SMB 63**	M	Over 23.5	50-59	3 permanent	33, 34, 43
**SMB 68**	M	Over 23.5	50-59	19 permanent	13, 14, 17, 18, 23, 24, 25, 31, 32, 34, 35, 37, 38, 42, 43, 44, 45, 47, 48
**SMB 84**	F	Over 23.5	50-59	0	N/A
**Total number of primary teeth present:**				**175**	
**Total number of Permanent teeth present:**				**343**	
**Grand Total of all tooth types present:**				**518**	

Notes: **Notes:** F= Female, M= male, U= undetermined. † Dental age range – "Over 23.5 years"= Estimation of dental age range is based on development and when this is complete. * FDI = Fédération Dentaire Internationale tooth identification notation system

References:

Anson, T.J., 2004. The bioarchaeology of the St. Mary's free ground burials: reconstruction of colonial South Australian lifeways, Thesis (Ph.D.)-University of Adelaide, Dept. of Anatomical Sciences, 2004.

AlQahtani, S.J., Hector, M.P., Liversidge, H.M., 2010. Brief communication: The London atlas of human tooth development and eruption, American Journal of Physical Anthropology 142, 481-490.

FDI World dental Federation, 2022. FDI Two-Digit Notation, FDI World Dental Federation, France.
