# Supplementary material for: The Oral Health of a Group of 19th Century South Australian Settlers in Relation to Their General Health and Compared with That of Contemporaneous Samples
Source: Dent J (Basel). 2023 Apr 7;11(4):99. doi: 10.3390/dj11040099 (PMC10136771; doi:10.3390/dj11040099)
Supplement: Supplementary file 1 [file dentistry-11-00099-s001.zip › Supplementary_Table S10. EH_number of defects per tooth.pdf]

Table S10. Enamel Hypoplastic Defects _ St Mary Cemetery Sample.

**St Mary's ID**	**Estimation of age** (skeletal & eruption findings) (years)	**Sex**	**Total number of teeth present**	**Permanent and/or primary dentition**	**Tooth type/s affected by EH defects** (anatomical tooth classification)	**Tooth type/s affected by EH defects** (*FDI notation numbers)	**Total number of teeth with EH defects**	**Percentage of teeth affected by EH defects**	**Type of EH defect/s present**	**Number of EH defects per tooth**	**Antemortem tooth loss** (*FDI notation numbers) *(evidence of healing or healed alveolar bone in the original position of the tooth)*
**SMB 58**	0-2	U	11	*All primary* teeth	Upp R & L Cent I Upp R & L Lat I, Low L Lat I, Upp R m1, Low L & R m1	51, 52, 54, 61, 62, 72, 74, 84	8	72%	Linear & pits	51, 61 = 1 line, 52, 62 = 1 line & 1 pit, 54, 72, 84 = group of multiple pits,	None
**SMB 11**	0-2	U	19	primary	Upp R & L Canines Low R & L Canines	53, 63, 73, 83	4	21%	Pits	53, 63, 73 & 83 = group of multiple pits	None
**SMB 04A**	3-5	U	19	primary	Low R & L Cent I Low R & L Lat I	71, 72, 81, 82	4	21%	Pits	71, 72, 81 & 82 = small pits in a line	None
**SMB 35**	6-9	U	11	primary	Upp L Canine	63	1	9%	Pits	63= 1 pit	None
**SMB 19**	6-9	U	22	*Mixed dentition:* 7 primary 15 Permanent	*Primary teeth*: Low R canine *Permanent teeth*: Upp R & L Cent I, Canine, P1, P2, Upp L Lat I. Low R Lat I.	12, 13, 14, 15, 21, 22, 42, 83	8	36%	Linear & pit	12= 1 pit, 13 = 1 pit, 14 = 1 pit, 15 = group of multiple pits, 21 = 1 line, 22 = 1 pit, 42 =1 pit, 83= 1 pit	None
**SMB 51**	10-15	U	16	1 primary 15 Permanent	*Permanent teeth*: Upp R Cent I, Canine, P1, M1, Upp L Lat I, Canine, P1, P2, M1, Low L P1, M1	12, 13, 14,16, 22, 23,24, 25, 26, 34, 36	11	69%	Linear & pit	12, 13 = 2 lines, 14, 24, 36 = 1 line & 1 pit, 16 = 1 line & a group of multiple pits, 22, 23, 26 = 2 lines, 25 = 2 lines & a group of multiple pits, 34 = 3 lines	None
**SMB 52B**	10-15	U	17	2 primary 15 permanent	*Permanent teeth*: Upp R Cent I, Lat I, Canine, M1, Upp L Cent I, Canine, M1, Low L Cent I, Lat I, Canine, M1, Low R Cent I, Lat I, Canine	11, 12, 13, 16, 21, 23, 26, 31, 32, 33, 36, 14, 42, 43	14	82%	Linear & pit	11, 12, 23, 26, 31, 32, 43 = 1 line, 13, 33, 42 = 2 lines, 16, 21, 36 = 1 pit, 41 = 1 line & 1 pit	None
**SMB 70**	10-15	U	8	2 primary 6 Permanent	*Primary teeth:* Upp R & L Canines *Permanent teeth*: Upp L Cent I, M1.	53, 21, 63, 26	4	50%	Linear & Pits	53, 63, 26 = a group of multiple pits, 21 = 3 lines & a group of multiple pits	None

**SMB 28**	10-15	U	26	*All Permanent*	Upp R Cent I, Lat I, P2, M1,Upp L Lat I, Canine, P2, M1, M2, Low L Cent I, Lat I, Canine, P1, P2, Low R Cent I, Lat I, Canine, P1, P2, M2	11, 12, 15, 16, 22, 23, 25, 26, 27, 31, 32, 33, 34, 35, 41, 42, 43, 44, 45, 47	20	77%	Linear & pit	11, 16, 27, 47 = a group of multiple pits (in 2 lines), 12, 22 = 2 lines, 15, 25, 41 = 1 pit 23, 43 = 3 lines, 26 = 1 line & a group of multiple pits, 31, 32, 33, 34, 35, 42, 44, 45 = 1 line,	None
**SMB 79**	16-19	U	25	Permanent	Upp R Canine, Upp L Canine, M2, Low R & L Canines	13, 23, 27, 33, 43	5	20%	Linear & pit	13 = 1 pit, 23, 27 = a group of multiple pits, 33 = 2 lines, 43 = 1 line & a group of multiple pits	36
**SMB 05**	20-29	F	6	Permanent	Low R Cent I, Lat I	41, 42	2	33%	Linear	41, 42 = 1 line	11, 12, 13, 14, 15, 16, 18, 21, 22, 23, 24, 25, 26, 27,28, 36, 37, 38, 45, 46, 47, 48
**SMB 53C**	30-39	F	9	Permanent	Upp R Cent I, Canine, P1, Upp L Cent I, Canine	11, 13, 14, 21, 23	5	56%	Linear & pit	11 = 2 lines & a group of pits, 13, 14 = 2 lines, 21 = 1 line, 23 = a groupof multiple pits	15, 16, 17, 18, 24, 25, 27, 28, 31, 35, 36, 37, 38, 41, 44, 47, 48
**SMB 66B**	30-39	F	17	permanent	Upp R P1, M2, Upp L Cent I, Canine, M2, Low L P1, Low R P1, P2	14, 17, 21, 23, 27, 34, 44, 45	8	47%	Pits	14, 17, 21, 23, 27, 44 & 45 = a group of multiple pits, 34 = 1 pit	11 (root only), 13, 16, 18, 26, 28, 36, 37, 38, 46, 47, 48
**SMB 73**	30-39	M	19	Permanent	Upp R Cent I, Lat I, Canine, M1, UppL Cent I, Canine, P2, Low L Cent I, Lat I, Canine, P1, Low R Cent I, Lat I, Canine, P1	11, 12, 13, 16, 21, 23, 25, 31, 32, 33, 34, 41, 42, 43, 44	15	79%	Linear & pits	11, 21, 31, 32, 41 & 42= 2 lines & a group of multiple pits, 12 = 1 line, 13, 23 33 & 43 = 1 line & a group of multiple pits, 16, 44 = 1 pit & a group of multiple pits, 25 = 1 pit	15, 17, 18, 24, 26, 27, 36, 37, 38, 46, 47, 48
**SMB 06**	40-49	M	24	Permanent	Upp R Cent I, Low L Cent I, Lat I, Low R Cent I, Lat I	12, 31, 32,41, 42	5	21%	Linear	12 = 3 lines, 31, 32, 41,42 = 1 line,	28, 34, 35, 36, 38, 48
**SMB 09**	40-49	M	22	Permanent	Upp R Cent I, Canine, Upp L Cent I, Lat I, Canine, Low L Lat I, Canine, Low R Canine	12, 13, 21, 22, 23, 32, 33, 43	8	36%	Linear & pit	12, 13, 21, 22, 23, 32, 33, 43 = 1 line	11, 27, 38
**SMB 57**	40-49	M	25	Permanent	Upp R Cent I, Lat I, Canine, P1, Upp L, M2, Low L Lat I, Canine, P2, Low R Lat I, Canine	11, 12, 13, 14, 27, 32, 33, 35, 42, 43	10	40%	Linear & pit	11, 12, 32, 33 & 42 = 2 lines, 13 = 1 line & a group of multiple pits, 14, 27 & 35= a group of multiple pits, 43 = 3 lines	25, 28
**SMB 72**	40-49	M	29	Permanent	Upp R Lat I, Canine, Upp L Canine, P1, Low L M3, Low R Canine, M3	12, 13, 23, 24, 38, 43, 48	7	24%	Pits	12, 38, 48= 1 pit, 13, 23, 24, 43 = a group of multiple pits (in a line)	
**SMB 83**	40-49	M	16	Permanent	Upp R Canine, Upp L Cent I, Canine, M2, M3, Low L	13, 21, 23, 27, 28, 33, 34, 42, 43	9	56%	Linear & pit	13, 23 = 2 lines, 21, 33, 34, 42 = 1 line, 27, 28 = a group of multiple pits,	16, 18, 26, 36, 45, 46

					Canine, P1, Low R Lat I, Canine					43 = 2 lines & a group of multiple pits	
**SMB 85**	40-49	M	2	Permanent	Low L Canine	33	1	50%	Linear & pit2	33 = 1 line & a group of multiple pits	11, 12, 13, 14, 15, 16, 17, 18, 23, 24, 25, 26, 27, 28, 31, 35, 36, 37, 38, 41, 43, 44, 45, 46, 47, 48
**SMB 23**	50-59	M	21	Permanent	Upp R M2, M3, Upp L Cent I, Low L Cent I, Low R Cent I, Lat I, Canine	17, 18, 21, 31, 32, 33, 41,42, 43	9	43%	Linear & pit	17, 18, 21, 33, 43 = a group of multiple pits, 31, 32, 41, 42 = 1 line	48
**SMB 59**	50-59	M	15	Permanent	Upp R Cent I, Lat I, Canine, Upp L Lat I, Canine, Low L P2, M3, Low R Cent I, Lat I, P1, M1	11, 12, 13, 21, 22, 23, 35, 38, 41, 42, 44, 46	12	80%	Linear & pit	11, 42 = 2 lines, 12, 13, 22, 23, 35, 38, 44, 46 = a group of multiple pits, 21 & 41 = 3 lines	14, 15, 24, 25, 47
**SMB 63**	50-59	M	3	Permanent	Low L Lat I, Canine	32, 43	2	67%	Pits	32 = a group of multiple pits, 43 = 1 pit	11, 12, 14, 15, 16, 17, 21, 22, 24, 25, 26, 27, 28, 35, 36, 37, 38, 41, 42, 44, 46, 47, 48
**SMB 68**	50-59	M	19	Permanent	Upp R P1, Upp L, Canine, Low L P2, Low R Canine, P1, P2	14, 23, 35, 43, 44, 45	6	42%	Pits	14, 23, 35, 43, 44, & 45 = a group of multiple pits	15, 16, 36, 41, 46
**Total number of primary teeth affected**							20				
**Total number of Permanent teeth affected**							158				
**Grand Total of teeth affected**							178				

Notes: **M** = male, **F** = female, **U** = undetermined sex. **EH** = Enamel hypoplastic defects. **Upp** = upper jaw (maxilla), **Low** = lower jaw (Mandible), **Cent.** = central, **Lat.** = lateral, **I** = incisor, **P1** = first premolar, **P2** = second premolar, **m1** = first *primary* molar, **M1**= first *permanent* molar, **m2** = second *primary* molar, **M2** = second *permanent* molar, **M3** = third permanent molar.

*A tooth may have more than one EH defect. **AMTL**= antemortem tooth loss = evidence of healing or the alveolar bone has healed in the original position of the tooth. **FDI** = Fédération Dentaire Internationale tooth identification notation system.
