# Supplementary material for: The Oral Health of a Group of 19th Century South Australian Settlers in Relation to Their General Health and Compared with That of Contemporaneous Samples
Source: Dent J (Basel). 2023 Apr 7;11(4):99. doi: 10.3390/dj11040099 (PMC10136771; doi:10.3390/dj11040099)
Supplement: Supplementary file 1 [file dentistry-11-00099-s001.zip › Supplementary_Table S2. LV Micro-CT Scan Settings.pdf]

Supplementary Material

Table S2. Large Volume Micro-CT. Settings used for scanning of the dentition in situ in the dentoalveolar complex of the human skulls from the St Mary's Cemetery individuals, using the Nikon XT H 225 ST Micro-CT system. In choosing the scanning settings, the transmitted signal intensity and source power settings were considered according to guidelines (Wearne et al 2022, du Plessis et al. 2017).

**Skeletal region LV Micro-CT scanned**	**St Mary's** ID	**Pixel size** (µm)	**Source voltage** (kV)	**Source Current** (µA)	**Source Power** (W)	**Rotation step**	**Exposure time** (seconds)	**Filter Type**	**Filter thickness** (mm)	**X-ray projections**	**Total acquisition time** (hours/minutes)
**Skull**	**SMB 73**	60	190	220	41	360º	1.42	Sn	0.1	3000	1 hr 10 mins
	**SMB 52B**	60	190	220	41	360º	1.42	Sn	0.1	3000	1 hr 10 mins
**Cranium only**	**SMB 82**	40	190	210	40	360º	1.42	Sn	0.1	3000	1 hr 10 mins
	**SMB 66B**	50	190	220	41	360º	1.42	Sn	0.1	3000	1 hr 10 mins
	**SMB 58**	35	190	184	35	360º	1.42	Sn	0.1	3000	1 hr 10 mins
	**SMB 4A**	55	190	220	41	360º	1.42	Sn	0.1	3000	1 hr 10 mins
**Mandible only**	**SMB 82**	18	190	68	12.9	360º	2	Al	0.5	3000	1 hr 40 mins
	**SMB 73**	13	190	68	12.9	360º	2	Al	0.5	3000	1 hr 40 mins
	**SMB 66B**	13	190	68	12.9	360º	2	Al	0.5	3000	1 hr 40 mins
	**SMB 58**	21	190	95	18	360º	2	Al	0.5	3000	1 hr 40 mins
	**SMB 52B**	22	190	68	12.9	360º	2	Al	0.5	3000	1 hr 40 mins
	**SMB 4A**	20	190	68	12.9	360º	2	Al	0.5	3000	1 hr 40 mins

References:

du Plessis, A., Broeckhoven, C., Guelpa, A., le Roux, S.G., 2017. Laboratory x-ray micro-computed tomography: a user guideline for biological samples, Gigascience 6, 1-11.

Wearne, L.S., Rapagna, S., Taylor, M., Perilli, E., 2022. Micro-CT scan optimisation for mechanical loading of tibia with titanium tibial tray: A digital volume correlation zero strain error analysis, Journal of the mechanical behavior of biomedical materials 134, 105336-105336.
