# Supplementary material for: The Oral Health of a Group of 19th Century South Australian Settlers in Relation to Their General Health and Compared with That of Contemporaneous Samples
Source: Dent J (Basel). 2023 Apr 7;11(4):99. doi: 10.3390/dj11040099 (PMC10136771; doi:10.3390/dj11040099)
Supplement: Supplementary file 1 [file dentistry-11-00099-s001.zip › Supplementary_Table S3. SV Micro-CT list_individuals, tooth type & number.pdf]

Table S3. Small Volume Micro-CT scanning of individual teeth associated with skulls from individuals of the St Mary's Cemetery sample. Additional SV Micro-CT scan settings information on page 2.

**St Mary's ID**	**Dental age range** (years & months)	**Chronological Age range** (years)	**Sex**	**Permanent or *primary* Tooth Type** *with the FDI notation number*	**Total number of teeth SV Micro-CT scanned**
**SMB 27B**	1-1.5 (+/- 3 mths)	0-2	U	*primary* Low L M1 (74)	**1**
**SMB 58**	1-1.5 (+/- 3 mths)	0-2	U	*primary* Low L m1 (74)	**1**
**SMB 82**	1-1.5 (+/- 3 mths)	0-2	U	*primary* Upp R *cent incisor (51)<br>*primary* Upp R lat incisor (52)<br>*primary* Upp L cent incisor (61)<br>*primary* Upp L lat incisor (62)<br>*primary* Low R cent incisor (71)<br>*primary* Low L cent incisor (81)	**6**
**SMB 4A**	3.5-4.5 (+/- 3 mths)	2-5	U	*primary* Upp R Lat Incisor (52)<br>*primary* Upp R Canine (53)<br>*primary* Upp L Cent Incisor (61)<br>*primary* Upp L Canine (63)	**4**
**SMB 19**	7.5- 8.5 (+/- 6 mths)	6-10	U	Permanent Low L M1 (36)	**1**
**SMB 70**	11.5-12.5 (+/- 6 mths)	8-9	U	*primary* Low R m1 (74)<br>Permanent Upp R M1 (16)	**2**
**SMB 52B**	10.5-11.5 (+/- 6 mths)	10-14	U	*primary* Low L m2 (75)<br>Permanent Upp R Cent Incisor (11)<br>Permanent Upp R lat Incisor (12)	**3**
**SMB 51**	10-11 (+/- 6 mths)	10-14	U	Permanent Low R M1 (46)	**1**
**SMB 28**	12-15 (+/- 6 mths)	12-16	U	Permanent Low L M1 (36)	**1**
**SMB 79**	15.5-16.5 (+/- 6 mths)	16-18	U (F?)	Permanent Upp L M1 (26)	**1**
**SMB 05**	Over 23.5	20-29	F	Permanent Low R Canine (43)	**1**
**SMB 53C**	Over 23.5	30-39	F	Permanent Upp R M1 (16)	**1**
**SMB 66B**	Over 23.5	30-39	F	Permanent Upp R lat incisor (12)<br>Permanent Upp R P1 (14)<br>Permanent Upp R P2 (15)<br>Permanent Upp R M2 (17)<br>Permanent Upp L canine (23)	**5**
**SMB 73**	Over 23.5	30-39	M	Permanent Upp R P1 (14)<br>Permanent Upp R Cent Incisor (11)<br>Permanent Upp R M1 (16)<br>Permanent Upp L Canine (23)<br>Permanent Upp L P2 (25)<br>Permanent Upp L M3 (28)	**6**
**SMB 6**	Over 23.5	40-49	M	Permanent Upp L Canine (23)	**1**
**SMB 72**	Over 23.5	40-49	M	Permanent Low R M2 (47)	**1**
**SMB 85**	Over 23.5	40-49	M	Permanent Low L canine (33)	**1**
**SMB 23**	Over 23.5	50-59	M	Permanent Low R M2 (47)	**1**
**SMB 63**	Over 23.5	50-59	M	Permanent Low R Lat Incisor (42)	**1**
**SMB 68**	Over 23.5	50-59	M	Permanent Low R M3 (48)	**1**
**SMB 83**	Over 23.5	50- 59	M	Permanent Upp R M3 (18)	**1**
				Total number of teeth SV Micro-CT scanned:	**41**

Table S3 Notes: M= male, F= female, U= undetermined sex, Low = lower/ mandibular tooth type, Upp = upper/ maxillary tooth type, L=left, R=right, Cent. /*cent. = central, (* lower case text denotes primary dentition, capitalised text denotes permanent teeth), Lat = lateral, P1 or P2 = first or second premolar, M1, M2 or M3 = first, second or third molar.

SV Micro-CT scan settings information:

Equipment: Bruker SkyScan 1276 (Bruker, 2022)

The desktop SV Micro-CT scanner was set at source voltage of 100 kV, source current of 200 µA, and camera binning of 4032 x 2688, with an aluminium and copper filter. The Nrecon software (Bruker, 2022), was used for the reconstruction of the scan data sets.

Reference:

Bruker, 2022. Position, Scan, Reconstruct and Analyze, Bruker. Micro Photonics Inc, 2020. Micro-CT Software: NRecon Reconstruction Software: Fast micro-CT reconstruction software., Micro Photonics Inc.
