# Supplementary material for: The Oral Health of a Group of 19th Century South Australian Settlers in Relation to Their General Health and Compared with That of Contemporaneous Samples
Source: Dent J (Basel). 2023 Apr 7;11(4):99. doi: 10.3390/dj11040099 (PMC10136771; doi:10.3390/dj11040099)
Supplement: Supplementary file 1 [file dentistry-11-00099-s001.zip › Supplementary_Table S4. Intra & Inter-Op Results_Statiscal Analysis Summary_.pdf]

Supplementary Material Table S4. Statistical Analysis Summary- Results from tests of intra- and inter-operator reliability for each of the methods assessed.

Test	Comparison	Summary Results		
		Agreement	AC2	Percentage agreement
Inventory				
Inter-operator	LV	Fair – Perfect	0.31 - 1.00	67 - 100
	Macro	Poor - Perfect	-0.01 - 1.00	56 - 100
	Radio	Substantial - Perfect	0.79 - 1.00	89 - 100
Intra-operator	LV	Perfect	All 1.00	All 100%
	Macro	Moderate - Perfect	0.53 - 1.00	67 – 100
	Radio	Substantial - Perfect	0.79 – 1.00	89 – 100
Tooth wear				
Inter-operator	LV	Fair - Perfect	0.20 – 1.00	63 – 100
	Macro	Moderate - Perfect	0.53 – 1.00	75 – 100
	Radio	Moderate - Perfect	0.47 – 1.00	75 – 100
Intra-operator	LV	Perfect	All 1.00	All 100%
	Macro	Almost perfect	0.81 – 100	88 – 100
	Radio	Substantial - Perfect	0.78 – 1.00	88 – 100
Alveolar status				
Inter-operator	LV	Poor – Perfect	-0.18 – 1.00	38 – 100
	Macro	Poor – Perfect	-0.43 – 1.00	38 – 100
Intra-operator	LV	Perfect	All 1.00	All 100%
	Macro	Fair – Perfect	0.20 – 1.00	50 – 100
Caries				
Inter-operator	LV	Poor – Perfect	-1.00 – 1.00	0 – 100
	Macro	Poor – Perfect	-0.45 – 1.00	0 – 100
	Radio	Poor – Perfect	-0.45 – 1.00	0 – 100
Intra-operator	LV	Fair – Perfect	0.20 – 1.00	50 – 100
	Macro	Fair – Perfect	0.27 – 1.00	50 – 100
	Radio	Fair – Perfect	0.20 – 1.00	50 – 100
Enamel hypoplastic defects				
Inter-operator	LV	Poor – Perfect	-0.57 – 1.00	0 – 100
	Macro	Fair – Perfect	0.20 – 1.00	50 – 100
Intra-operator	LV	Fair – Perfect	0.20 – 1.00	50 – 100
	Macro	Fair – Perfect	0.27 – 1.00	50 – 100
Periodontal disease				
			**ICC**	
Inter-operator	LV	Poor – Excellent	0.23 – 1.00	
	Macro	Poor – Excellent	0.21 – 1.00	
Intra-operator	LV	Poor – Excellent	0.46 – 1.00	
	Macro	Poor – Excellent	0.00 – 1.00	

Results are presented as AC1/AC2 with 95% confidence interval (CI) and percentage agreement for non-continuous data; ICC with 95% CI for continuous data. Interpretation of AC1 and AC2 was <0 poor agreement; 0 – 0.2 slight agreement; 0.21 – 0.4 fair agreement; 0.41 – 0.6 moderate agreement; 0.61 – 0.8 substantial agreement; >0.8 almost perfect agreement (Landis and Koch, 1977). Interpretation of ICC values was <0.50 poor agreement; 0.50-0.75

moderate agreement; 0.75-0.90 good agreement; >0.90 excellent agreement (Koo and Li, 2016). All analyses were performed using Stata v17 (StataCorp, 2022).

Supplementary Material: Results - Reproducibility

Table S4. (above), presents a summary of the results from tests of intra- and inter-operator reliability for each of the methods assessed. Additional information from the statistical analysis can be found in the supplementary material as Table S5 and the raw data as Data_S1_Gurr et al. 2022_RAW DATA_S1_EXCEL_.

Due to the small sample size, many tests achieved perfect agreement resulting in all summary ranges including perfect or excellent agreement. The LV Micro-CT method achieved better agreement than the Macroscopic and Radiographic techniques when assessing intra-operator agreement for inventory measures, tooth wear, and alveolar status (perfect agreement for all, compared with moderate to perfect (Macroscopic) or substantial to perfect (Radiographic); almost perfect (Macroscopic) or substantial to perfect (Radiographic); and fair to perfect (Macroscopic); for inventory, tooth wear and alveolar status, respectively). However, for the inventory and tooth wear measures the inter-operator agreement was better when using the Macroscopic or Radiographic techniques (poor to perfect (Macroscopic) and substantial to perfect (Radiographic) compared with fair to perfect (LV Micro-CT); and moderate to perfect (both Macroscopic and Radiographic) compared fair to perfect (LV Micro-CT); for inventory and tooth wear, respectively). For alveolar status the inter-operator agreement was the same for both LV Micro-CT and Macroscopic.

The LV Micro-CT method achieved the same level of agreement for all other measures when assessing both inter- and intra-operator as the other techniques. It should be noted though that the percentage agreement was very similar, between 81 and 100% agreement for LV Micro-CT and 88 to 100% agreement for Macroscopic.

References:

The oral health of a group of 19th Century South Australian settlers in relation to their general health and compared with that of contemporaneous samples Gurr et al., 2023.

Koo, TK, & Li, MY 2016. A Guideline of Selecting and Reporting Intraclass Correlation Coefficients for Reliability Research. *Journal of chiropractic medicine, 15*(2), 155-163. DOI: 10.1016/j.jcm.2016.02.012

Landis, JR, & Koch, GG 1977. The Measurement of Observer Agreement for Categorical Data. *Biometrics, 33*(1), 159-174. DOI:10.2307/2529310

StataCorp LLC. (2022). Stata, Home: Products: New in Stata. Retrieved from https://www.stata.com/new-in-stata/
