# Supplementary material for: The Oral Health of a Group of 19th Century South Australian Settlers in Relation to Their General Health and Compared with That of Contemporaneous Samples
Source: Dent J (Basel). 2023 Apr 7;11(4):99. doi: 10.3390/dj11040099 (PMC10136771; doi:10.3390/dj11040099)
Supplement: Supplementary file 1 [file dentistry-11-00099-s001.zip › Supplementary_Table S5. Additional Info_Intra & Inter-Op Results_Statiscal Analysis_.pdf]

Supplementary Material Table S5. Additional Information from the Statistical Analysis of intra- and inter-operator reliability for each of the methods assessed.

Test	Comparison	Surface/Method	Summary Results		
			Agreement	AC2	Percentage agreement
		Caries			
Inter-operator	LV	Radiolucency	Substantial – Perfect	0.71 – 1.00	79 – 100
		Caries - Present	Poor – Perfect	-1.00 – 1.00	0 – 100
		Caries on CEJ	Poor – Perfect	-0.33 – 1.00	0 – 100
		Caries above CEJ	Poor – Perfect	-0.45 – 1.00	0 – 100
		Caries below CEJ	Poor – Perfect	-0.33 – 1.00	0 – 100
		Caries on distal	Poor – Perfect	-0.33 – 1.00	0 – 100
		Caries on labial/buccal	Perfect	All 1.00	All 100%
		Caries on lingual	Perfect	All 1.00	All 100%
		Caries on mesial	Poor – Perfect	-0.45 – 1.00	0 – 100
		Caries on occlusal	Perfect	All 1.00	All 100%
	Macro	Caries - Present	Fair – Perfect	0.20 – 1.00	50 – 100
		Caries on CEJ	Poor – Perfect	-0.45 – 1.00	0 – 100
		Caries above CEJ	Poor – Perfect	-0.45 – 1.00	0 – 100
		Caries below CEJ	Fair – Perfect	0.27 – 1.00	50 – 100
		Caries on distal	Fair – Perfect	0.27 – 1.00	50 – 100
		Caries on labial/buccal	Poor – Perfect	-0.33 – 1.00	0 – 100
		Caries on lingual	Fair – Perfect	0.33 – 1.00	50 – 100
		Caries on mesial	Poor – Perfect	-0.45 – 1.00	0 – 100
		Caries on occlusal	Fair – Perfect	0.27 – 1.00	50 – 100
	Radio	Radiolucency	Substantial – Perfect	0.73 – 1.00	83 – 100
		Caries - Present	Poor – Perfect	-0.60 – 1.00	0 – 100
		Caries on distal	Poor – Perfect	-0.23 – 1.00	0 – 100
		Caries on mesial	Fair – Perfect	0.27 – 1.00	50 – 100
Intra-operator	LV	Radiolucency	Substantial – Perfect	0.77 – 1.00	83 – 100
		Caries - Present	Fair – Perfect	0.20 – 1.00	50 – 100

		Caries on CEJ	Fair – Perfect	0.38 – 1.00	50 – 100
		Caries above CEJ	Fair – Perfect	0.38 – 1.00	50 – 100
		Caries below CEJ	Fair – Perfect	0.27 – 1.00	50 – 100
		Caries on distal	Fair – Perfect	0.27 – 1.00	50 – 100
		Caries on labial/buccal	Perfect	All 1.00	All 100%
		Caries on lingual	Fair – Perfect	0.38 – 1.00	50 – 100
		Caries on mesial	Fair – Perfect	0.38 – 1.00	50 – 100
		Caries on occlusal	Fair – Perfect	0.38 – 1.00	50 – 100
	Macro	Radiolucency	Moderate – Perfect	0.53 – 1.00	67 – 100
		Caries - Present	Fair – Perfect	0.27 – 1.00	50 – 100
		Caries on CEJ	Fair – Perfect	0.27 – 1.00	50 – 100
		Caries above CEJ	Fair – Perfect	0.27 – 1.00	50 – 100
		Caries below CEJ	Fair – Perfect	0.27 – 1.00	50 – 100
		Caries on distal	Fair – Perfect	0.27 – 1.00	50 – 100
		Caries on labial/buccal	Fair – Perfect	0.27 – 1.00	50 – 100
		Caries on lingual	Fair – Perfect	0.38 – 1.00	50 – 100
		Caries on mesial	Fair – Perfect	0.27 – 1.00	50 – 100
		Caries on occlusal	Fair – Perfect	0.27 – 1.00	50 – 100
	Radio	Radiolucency	Substantial - Perfect	0.66 – 1.00	75 – 100
		Caries - Present	Fair – Perfect	0.20 – 1.00	50 – 100
		Caries on distal	Fair – Perfect	0.27 – 1.00	50 – 100
		Caries on mesial	Fair – Perfect	0.27 – 1.00	50 – 100
