# Supplementary material for: The Oral Health of a Group of 19th Century South Australian Settlers in Relation to Their General Health and Compared with That of Contemporaneous Samples
Source: Dent J (Basel). 2023 Apr 7;11(4):99. doi: 10.3390/dj11040099 (PMC10136771; doi:10.3390/dj11040099)
Supplement: Supplementary file 1 [file dentistry-11-00099-s001.zip › Supplementary_Table S6. Miles (1962) Tooth wear_functional age of tooth & Predicted age range of subject.pdf]

Table S6. Adults of St Mary's Cemetery sample. Assessment of the functional age of each molar and the predicted age of the subject based on tooth wear scores set out by **Miles (1962).** *Notes for abbreviations follow this table.

St Mary's ID	Age range (skeletal /eruption findings) (years)	Miles (1962) tooth wear (years)	Jaw and tooth identification, including FDI notation numbers:											
			MAXILLA						MANDIBLE					
			Upp R **M1** 16	Upp R **M2** 17	Upp R **M3** 18	Upp L **M1** 26	Upp L **M2** 27	Upp L **M3** 28	Low L **M1** 36	Low L **M2** 37	Low L **M3** 38	Low R **M1** 46	Low R **M2** 47	Low R **M3** 48
**SMB 53C**	30-39	Functional age of teeth	**30**	AMTL	AMTL	AMTL	AMTL	AMTL	AMTL	AMTL	AMTL	**24**	AMTL	AMTL
		Predicted age of subject	**36**	n/a	n/a	n/a	n/a	n/a	n/a	n/a	n/a	**30**	n/a	n/a
**SMB 66B**	30-39	Functional age	AMTL	**7**	AMTL	AMTL	12	AMTL	AMTL	AMTL	AMTL	AMTL	AMTL	AMTL
		Predicted age	n/a	**18**	n/a	n/a	26	n/a	n/a	n/a	n/a	n/a	n/a	n/a
**SMB 73**	30-39	Functional age	**12**	AMTL	AMTL	AMTL	AMTL	**7**	AMTL	AMTL	AMTL	AMTL	AMTL	AMTL
		Predicted age	**18**	n/a	n/a	n/a	n/a	**24**	n/a	n/a	n/a	n/a	n/a	n/a
**SMB 06**	40-49	Functional age	**30**	**13**	AMTL	**36**	**32**	PMTL	AMTL	**28**	Not erupted	**36**	**20**	Not erupted
		Predicted age	**36**	**27**	n/a	**42**	**45**	n/a	n/a	**40**	n/a	**42**	**31**	n/a
**SMB 09**	40-49	Functional age	**18**	PMTL	PMTL	**12**	PMTL	PMTL	**15**	**7**	AMTL	**12**	**6.5**	**7**
		Predicted age	**24**	n/a	n/a	**18**	n/a	n/a	**21**	**19**	n/a	**18**	**18**	**25**
**SMB 57**	40-49	Functional age	**27**	**25**	PMTL	**24**	**25**	AMTL	**16**	**16**	PMTL	**23**	**20**	**7**
		Predicted age	**35**	**35**	n/a	**30**	**35**	n/a	**22**	**28**	n/a	**30**	**32**	**25**
**SMB 72**	40-49	Functional age	**12**	**5**	**14**	**12**	**5**	AMTL	**12**	**16**	**10**	**12**	**7**	**7**
		Predicted age	**18**	**17**	**26**	**18**	**17**	n/a	**18**	**27**	**25**	**18**	**18**	**24**
**SMB 83**	40-49	Functional age	AMTL	AMTL	**32**	AMTL	**26**	**24**	AMTL	**26**	**42**	AMTL	**26**	**35**
		Predicted age	n/a	n/a	**50**	n/a	**38**	**42**	n/a	**38**	**60**	n/a	**38**	**53**
**SMB 23**	50-59	Functional age	PMTL	PMTL	**28**	PMTL	PMTL	**7**	AMTL	**10**	AMTL	AMTL	**7**	AMTL
		Predicted age	n/a	n/a	**46**	n/a	n/a	**25**	n/a	**22**	n/a	n/a	**19**	n/a
**SMB 59**	50-59	Functional age	AMTL	PMTL	PMTL	AMTL	PMTL	PMTL	PMTL	**7**	**7**	**30**	**13**	AMTL
		Predicted age	n/a	n/a	n/a	n/a	n/a	n/a	n/a	**19**	**25**	**36**	**25**	n/a
**SMB 68**	50-59	Functional age	AMTL	**46**	**49**	PMTL	PMTL	PMTL	AMTL	**13**	**7**	AMTL	**20**	**24**
		Predicted age	n/a	**58**	**67**	n/a	n/a	n/a	n/a	**25**	**24**	n/a	**32**	**42**

***Notes: Upp** = Upper jaw (maxilla), **Low** = Lower jaw (mandible), **R** = Right side of the jaw, **L** = left side of the jaw. **FDI** = Fédération Dentaire Internationale tooth identification notation system. **AMTL** = Antemortem tooth loss. **PMTL**= Post-mortem tooth loss. **N/A** = not applicable. Not erupted = the tooth had not erupted through the alveolar bone. The alveolar bone did not show signs of healing and dental radiographs confirmed that the tooth was *or* was not within the bone. '*Functional age of tooth'* was scored by the wear on the occlusal surface of the permanent **M1** (first molar), **M2** (second molar), and **M3** (third molar) following Miles's (1962) system. '*Predicted age of subject'* (individual person) following Miles's (1962) scoring system.
