# Supplementary material for: The Oral Health of a Group of 19th Century South Australian Settlers in Relation to Their General Health and Compared with That of Contemporaneous Samples
Source: Dent J (Basel). 2023 Apr 7;11(4):99. doi: 10.3390/dj11040099 (PMC10136771; doi:10.3390/dj11040099)
Supplement: Supplementary file 1 [file dentistry-11-00099-s001.zip › Supplementary_Table S7. a-c_MOLNAR ( 1971) St Mary's Adults_.pdf]

Table S7a. Summary of findings for St Mary's Cemetery adults using Molnar's (1971) categories of tooth wear.

St Mary's Burial ID	Age range (years	Sex	Total number of permanent teeth present	Number of maxillary teeth	Number of mandibular teeth	Number of teeth with Cat 1-3	Number of teeth with Cat. 4	Number of teeth with Cat.5	Number of teeth with Cat. 6	Number of teeth with Cat. 7	Number of teeth with Cat. 8
**SMB 05**	20-29	F	6	0	6	4	2	0	0	0	0
**SMB 53C**	30-39	F	9	6	3	4	2	3	0	0	0
**SMB 66B**	30-39	F	17	8	9	9	7	1	0	0	0
**SMB 73**	30-39	M	19	9	10	6	12	1	0	0	0
**SMB 06**	40-49	M	25	13	12	1	10	8	5	1	0
**SMB 09**	40-49	M	22	9	13	14	4	4	0	0	0
**SMB 57**	40-49	M	25	11	14	3	10	12	0	0	0
**SMB 61**	40-49	F	6	2	4	4	1	0	0	1	0
**SMB 72**	40-49	M	30	15	15	16	11	3	0	0	0
**SMB 78**	40-49	F	4	2	2	1	0	2	1	0	0
**SMB 83**	40-49	M	15	7	8	2	7	4	2	0	0
**SMB 85**	40-49	M	2	0	2	0	0	2	0	0	0
**SMB 14**	50-59	M	2	1	1	0	1	0	1	0	0
**SMB 23**	50-59	M	21	10	11	8	6	6	0	0	1
**SMB 59**	50-59	M	16	6	10	5	5	1	4	1	0
**SMB 63**	50-59	M	3	0	3	0	1	2	0	0	0
**SMB 68**	50-59	M	19	7	12	5	4	8	2	0	0
**TOTAL**			**241**	**106**	**135**	**82**	**83**	**57**	**15**	**3**	**1**

Table S7b. Molnar's (1971) Category of tooth wear assigned to the *maxillary* dentition of the adults from the St Mary's sample.

St Mary's Burial ID	Age range (years	Sex	Jaw and permanent tooth identification, including FDI numbers<br>**Maxilla**															
			Upp R Cent. Incisor **11**	Upp R Lat Incisor **12**	Upp R Canine **13**	Upp R P1 **14**	Upp R P2 **15**	Upp R M1 **16**	Upp R M2 **17**	Upp R M3 **18**	Upp L Cent Incisor **21**	Upp L Lat Incisor **22**	Upp L Canine **23**	Upp L P1 **24**	Upp L_ P2 **25**	Upp L M1 **26**	Upp L M2 **27**	Upp L M3 **28**
**SMB 05**	20-29	F	N/A	N/A	N/A	N/A	N/A	N/A	N/A	N/A	N/A	N/A	N/A	N/A	N/A	N/A	N/A	N/A
**SMB 53C**	30-39	F	5	N/A	4	N/A	N/A	5	N/A	N/A	5	N/A	2	2	N/A	N/A	N/A	N/A
**SMB 66B**	30-39	F	N/A	3	N/A	3	2	N/A	2	N/A	5	3	3	N/A	N/A	N/A	4	N/A
**SMB 73**	30-39	M	4	4	3	N/A	N/A	2	N/A	N/A	4	4	4	N/A	4	N/A	N/A	2
**SMB 06**	40-49	M	5	4	3	5	5	6	6	N/A	N/A	4	5	4	4	6	6	N/A
**SMB 09**	40-49	M	N/A	5	4	N/A	N/A	2	N/A	N/A	5	4	3	3	3	3	N/A	N/A
**SMB 57**	40-49	M	5	5	5	4	4	4	5	N/A	N/A	5	N/A	3	N/A	4	5	N/A
**SMB 61**	40-49	F	N/A	N/A	N/A	7	N/A	N/A	N/A	N/A	N/A	N/A	N/A	N/A	1	N/A	N/A	N/A
**SMB 72**	40-49	M	5	3	4	2	2	3	4	4	5	4	4	3	3	4	3	N/A
**SMB 78**	40-49	F	N/A	N/A	5	2	N/A	N/A	N/A	N/A	N/A	N/A	N/A	N/A	N/A	N/A	N/A	N/A
**SMB 83**	40-49	M	N/A	N/A	5	4	N/A	N/A	N/A	5	4	N/A	4	3	N/A	N/A	4	3
**SMB 85**	40-49	M	N/A	N/A	N/A	N/A	N/A	N/A	N/A	N/A	N/A	N/A	N/A	N/A	N/A	N/A	N/A	N/A
**SMB 14**	50-59	M	N/A	N/A	N/A	N/A	N/A	N/A	N/A	N/A	N/A	N/A	6	N/A	N/A	N/A	N/A	N/A
**SMB 23**	50-59	M	N/A	N/A	5	4	2	N/A	2	2	5	5	5	N/A	N/A	N/A	2	8
**SMB 59**	50-59	M	4	4	5	N/A	N/A	N/A	N/A	N/A	4	4	6	N/A	N/A	N/A	N/A	N/A
**SMB 63**	50-59	M	N/A	N/A	N/A	N/A	N/A	N/A	N/A	N/A	N/A	N/A	N/A	N/A	N/A	N/A	N/A	N/A
**SMB 68**	50-59	M	N/A	N/A	5	5	N/A	N/A	6	6	N/A	N/A	5	4	4	N/A	N/A	N/A

Table S7c. Molnar's (1971) Category of tooth wear assigned to the *mandibular* dentition of the adults from the St Mary's sample.

St Mary's Burial ID	Age range (years)	Sex	Jaw and permanent tooth identification, including FDI numbers<br>**Mandible**<br>Low L Cent Incisor **31**	Low L Lat Incisor **32**	Low L Canine **33**	Low L P1 **34**	Low L_ P2 **35**	Low L M1 **36**	Low L M2 **37**	Low L M3 **38**	Low R Cent. Incisor **41**	Low R Lat Incisor **42**	Low R Canine **43**	Low R P1 **44**	Low R P2 **45**	Low R M1 **46**	Low R M2 **47**	Low R M3 **48**
**SMB 05**	20-29	F	4	N/A	1	2	N/A	N/A	N/A	N/A	3	4	N/A	2	N/A	N/A	N/A	N/A
**SMB 53C**	30-39	F	N/A	N/A	N/A	3	N/A	N/A	N/A	N/A	N/A	4	N/A	N/A	N/A	2	N/A	N/A
**SMB 66B**	30-39	F	4	4	4	3	N/A	N/A	N/A	N/A	4	4	4	3	3	N/A	N/A	N/A
**SMB 73**	30-39	M	4	4	5	2	4	N/A	N/A	N/A	4	4	3	3	4	N/A	N/A	N/A
**SMB 06**	40-49	M	5	4	4	5	N/A	N/A	7	N/A	4	4	5	4	4	6	5	N/A
**SMB 09**	40-49	M	5	5	3	3	3	4	4	N/A	N/A	N/A	3	2	2	3	3	3
**SMB 57**	40-49	M	N/A	5	5	5	4	4	4	N/A	5	5	5	4	3	4	4	3
**SMB 61**	40-49	F	N/A	3	4	N/A	2	N/A	N/A	N/A	N/A	2	N/A	N/A	N/A	N/A	N/A	N/A
**SMB 72**	50-59	M	4	4	5	2	2	3	3	2	N/A	4	4	2	2	4	3	2
**SMB 78**	40-49	F	N/A	5	6	N/A	N/A	N/A	N/A	N/A	N/A	N/A	N/A	N/A	N/A	N/A	N/A	N/A
**SMB 83**	40-49	M	N/A	N/A	5	5	N/A	N/A	5	6	N/A	4	4	N/A	N/A	N/A	4	6
**SMB 85**	40-49	M	N/A	N/A	5	5	N/A	N/A	N/A	N/A	N/A	N/A	N/A	N/A	N/A	N/A	N/A	N/A
**SMB 14**	50-59	M	N/A	N/A	N/A	N/A	4	N/A	N/A	N/A	N/A	N/A	N/A	N/A	N/A	N/A	N/A	N/A
**SMB 23**	50-59	M	5	5	4	4	2	N/A	2	N/A	4	4	4	3	N/A	N/A	2	N/A
**SMB 59**	50-59	M	N/A	N/A	N/A	6	7	6	N/A	2	3	3	N/A	6	4	2	N/A	2
**SMB 63**	50-59	M	N/A	N/A	5	5	N/A	N/A	N/A	N/A	N/A	N/A	4	N/A	N/A	N/A	N/A	N/A
**SMB 68**	50-59	M	5	4	N/A	3	3	N/A	3	3	N/A	5	4	5	2	N/A	5	5

Notes: M = male, **F** = female. A category was assigned to each tooth following **Molnar's (1971) categories of occlusal tooth wear**. A higher category implies more tooth wear.Category 1 =1, Category 2 =2, Category 3 =3, Category 4 =4, Category 5 =5, Category 6 =6, Category 7 = 7, Category 8 = 8. **Upp** = upper, **Low** = lower, **Cent.** = central, **Lat**. = lateral, **P1** = first premolar, **P2** = second premolar, **M1** = first permanent molar, **M2** = second permanent molar, **M3** = third permanent molar. **N/A** = not applicable – tooth either lost antemortem or post-mortem and could not be observed.
