# Supplementary material for: The Oral Health of a Group of 19th Century South Australian Settlers in Relation to Their General Health and Compared with That of Contemporaneous Samples
Source: Dent J (Basel). 2023 Apr 7;11(4):99. doi: 10.3390/dj11040099 (PMC10136771; doi:10.3390/dj11040099)
Supplement: Supplementary file 1 [file dentistry-11-00099-s001.zip › Supplementary_Table S8. a,b_Carious Lesions_locations_ (AutoRecovered).pdf]

The oral health of a group of 19th Century South Australian settlers in relation to their general health and compared with that of contemporaneous samples. Gurr et al., 2023.

Table S8a. St Mary's Cemetery Sample. Location of carious lesions on the surface/s of the teeth.

**St Mary's ID**	Estimation of age range skeletal & eruption findings (years)	Sex	*Total number of teeth present	**Caries *above* the CEJ	**Caries *on* the CEJ	**Caries *below* the CEJ	Location of caries: Mesial	Location of caries: Distal	Location of caries: Occlusal	Location of caries: Labial/ Buccal	Location of caries: Lingual/ Palatal	Total number of Carious lesions
**SMB 19**	6-10	U	22	2	1	0	2	0	0	1	0	3
**SMB 70**	8-12	U	8	2	1	0	1	1	1	0	0	3
**SMB 28**	10-14	U	26	2	0	0	0	0	1	1	0	2
**SMB 79**	15-18	U	25	11	1	0	8	4	0	1	0	13
**SMB 05**	20-29	F	6	5	1	3	2	3	0	1	2	8
**SMB 53C**	30-39	F	9	12	0	0	8	3	1	0	0	12
**SMB 66B**	30-39	F	17	7	7	2	6	4	2	3	1	16
**SMB 73**	30-39	M	19	5	31	3	12	14	5	4	4	39
**SMB 06**	40-49	M	24	9	1	9	3	1	5	4	6	19
**SMB 09**	40-49	M	22	11	0	0	5	6	0	0	0	11
**SMB 57**	40-49	M	25	4	5	3	2	1	3	6	0	12
**SMB 61**	40-49	F	7	5	0	0	0	3	1	1	0	5
**SMB 72**	40-49	M	29	5	8	2	2	0	5	5	3	15
**SMB 78**	40-49	M	4	0	3	0	2	1	0	0	0	3
**SMB 83**	40-49	M	16	7	0	0	1	2	4	0	0	7
**SMB 85**	40-49	M	2	1	0	0	1	0	0	0	0	1
**SMB 14**	50-59	M	2	1	0	0	0	0	0	0	1	1
**SMB 23**	50-59	M	21	15	34	5	16	16	4	10	8	54
**SMB 59**	50-59	M	15	2	6	2	2	2	0	6	0	10
**SMB 63**	50-59	M	3	2	1	0	2	0	0	1	0	3
**SMB 68**	50-59	M	19	6	8	0	2	4	2	6	0	14
		**TOTAL**	**321**	**114**	**108**	**29**	**77**	**65**	**34**	**50**	**25**	**251**

Note: *A tooth may have more than one carious lesion present. † CEJ= Cementoenamel Junction. **More than one carious lesion may be present *above* and/ or *on* and /or *below* the CEJ.

Table S8b. St Mary’s Cemetery Sample. Number of carious lesions involving either the enamel only, the enamel and dentine, or the enamel, dentine and pulp.

**St Mary’s ID**	Estimation of age range skeletal & eruption findings (years)	Sex	*Total number of teeth present		Total number of Carious lesions present		Carious lesions involving the enamel	Carious lesions involving the enamel & dentine	Carious lesions involving the enamel, dentine & pulp
**SMB 19**	6-10	U	22		3		3	0	0
**SMB 70**	8-12	U	8		3		3	0	0
**SMB 28**	10-14	U	26		2		2	0	0
**SMB 79**	15-18	U	25		12		11	1	0
**SMB 05**	20-29	F	6		9		7	2	0
**SMB 53C**	30-39	F	9		12		11	1	0
**SMB 66B**	30-39	F	17		16		14	2	0
**SMB 73**	30-39	M	19		39		39	0	0
**SMB 06**	40-49	M	24		19		19	0	0
**SMB 09**	40-49	M	22		11		11	0	0
**SMB 57**	40-49	M	25		12		9	3	0
**SMB 61**	40-49	F	7		5		1	2	2
**SMB 72**	40-49	M	29		15		15	0	0
**SMB 78**	40-49	M	4		3		3	0	0
**SMB 83**	40-49	M	16		7		7	0	0
**SMB 85**	40-49	M	2		1		1	0	0
**SMB 14**	50-59	M	2		1		1	0	0
**SMB 23**	50-59	M	21		54		50	2	2
**SMB 59**	50-59	M	15		10		9	1	0
**SMB 63**	50-59	M	3		3		2	0	1
**SMB 68**	50-59	M	19		14		14	0	0
		**TOTAL**	**321**		**251**		**232**	**14**	**5**

Note: *A tooth may have more than one carious lesion present.
