# Supplementary material for: The Oral Health of a Group of 19th Century South Australian Settlers in Relation to Their General Health and Compared with That of Contemporaneous Samples
Source: Dent J (Basel). 2023 Apr 7;11(4):99. doi: 10.3390/dj11040099 (PMC10136771; doi:10.3390/dj11040099)
Supplement: Supplementary file 1 [file dentistry-11-00099-s001.zip › Supplementary_Table S9. Calculus_Adults_St Mary's.pdf]

Table S9. Dental calculus – an indication of the number, and percentage of teeth affected by calculus with the location and severity of the deposit for the adults of the St Mary's Cemetery sample.

**St Mary's Burial I/D**	**Sex**	Estimation of age range skeletal & eruption findings (years)	Total number of permanent teeth present	**Number of Teeth with calculus**	**Percentage of teeth with calculus**	**Calculus Present**<br><br>Tooth-type with calculus present<br><br>FDI notation number/s	**Calculus Location**<br><br>1 = enamel surface only<br>2 = Root*<br><br>(Brothwell, 1963; Connell and Rauxloh, 2003; Powers, 2012)	**Calculus Severity**<br><br>1 = small (slight)<br>2 = medium<br>3 = large (heavy/ considerable)<br><br>(Brothwell, 1963; Connell and Rauxloh, 2003; Powers, 2012)
**SMB 05**	F	20-29	6	6	100%	32, 35 41, 42, 43, 44	All teeth = enamel	35, 44 = small (slight),<br>32, 41, 42, & 43 = medium
**SMB 53 C**	F	30-39	9	1	11%	42	42 = root	42 = small
**SMB 66 B**	F	30-39	17	6	35%	31, 32, 41, 42, 43, 44	All teeth = root	All teeth = small
**SMB 73**	M	30-39	19	0	0	N/A	N/A	N/A
**SMB 06**	M	40-49	24	14	58%	12, 16, 17, 23, 25, 26, 27, 32, 33, 37, 41, 44, 45, 47	12, 17, 23, 26, 32, 33, 37, 45 = enamel,<br>16, 25, 27, 47 = root	12, 17, 23, 25, 26, 27, 32, 33, 37, 44, 45, 47 = small,<br>16 = medium
**SMB 09**	M	40-49	22	15	68%	12, 13, 16, 26, 27, 31, 32, 36, 37, 43, 44, 45, 46, 47, 48	12, 13, 16, 22, 23, 24, 26, 31, 32, 36, 43, 44, 45, 46, 47, 48 = enamel,<br>26, 37 = root	All teeth = small
**SMB 57**	M	40-49	25	14	56%	15, 16, 17, 18, 22, 26, 27, 32, 33, 34, 35, 36, 44, 45	All teeth = enamel	All teeth = small
**SMB 61**	F	40-49	7	3	43%	32, 42, 44	32 & 42 = enamel,<br>44 = root	All teeth = small
**SMB 72**	M	40-49	29	2	7%	42, 43	42 & 43 = enamel	All teeth = small
**SMB 78**	M	40-49	4	4	100%	13, 14, 34, 35	All teeth = enamel	All teeth = small
**SMB 83**	M	40-49	16	14	88%	13, 14, 17, 23, 24, 27, 28, 33, 34, 37, 42, 43, 47, 48	14, 17, 23, 24, 27, 28, 42, 43, 47,48 = enamel,<br>13, 33, 34, 37, 42 = root	13, 14, 17, 28, 33, 34, 37 = small,<br>23, 24, 27, 42, 43, 47, 48 = medium
**SMB 85**	M	40-49	2	0	0	N/A	N/A	N/A
**SMB 14**	M	50-59	2	0	0	N/A	N/A	N/A
**SMB 23**	M	50-59	21	0	0	N/A	N/A	N/A
**SMB 59**	M	50-59	15	1	7%	46	46 = enamel	46 = small
**SMB 63**	M	50-59	3	0	0	N/A	N/A	N/A
**SMB 68**	M	50-59	19	0	0	N/A	N/A	N/A

The oral health of a group of 19th Century South Australian settlers in relation to their general health and compared with that of contemporaneous samples. Gurr et al., 2023.

Notes: F = female, M = male. † Dental eruption age range – "Over 23.5 years"= Estimation of dental age range is based on development and when this is complete. FDI = Fédération Dentaire Internationale tooth identification notation system. *Guidelines, system, and criteria followed as set out by Brothwell, 1963; Connell and Rauxloh, 2003; Powers, 2012, of the Museum of London.

References:

Anson, T.J., 2004. The bioarchaeology of the St. Mary's free ground burials: reconstruction of colonial South Australian lifeways, Thesis (Ph.D.)-University of Adelaide, Dept. of Anatomical Sciences, 2004.

AlQahtani, S.J., Hector, M.P., Liversidge, H.M., 2010. Brief communication: The London atlas of human tooth development and eruption, American Journal of Physical Anthropology 142, 481-490.

Brothwell, D.R. 1963. *Digging up Bones: the excavation, treatment and study of the human skeletal remains*. British Museum Natural History: London.

Connell, B, and Rauxloh, P. 2003. *A Rapid Method for Recording Human Skeletal Data*. Museum of London: London.

FDI World Dental Federation, 2022. FDI Two-Digit Notation, FDI World Dental Federation, France.

Powers, N. 2012. *Human Osteology Method Statement*. Museum of London: London.
